# Uptake of Etoposide in CT-26 Cells of Colorectal Cancer Using Folate Targeted Dextran Stearate Polymeric Micelles

**DOI:** 10.1155/2014/708593

**Published:** 2014-02-10

**Authors:** Jaleh Varshosaz, Farshid Hassanzadeh, Hojjat Sadeghi-Aliabadi, Farzin Firozian

**Affiliations:** ^1^Department of Pharmaceutics, Novel Drug Delivery Systems Research Center, Faculty of Pharmacy, Isfahan University of Medical Sciences, Isfahan 81745-359, Iran; ^2^Department of Medicinal Chemistry, Faculty of Pharmacy, Isfahan University of Medical Sciences, Isfahan 81746-73461, Iran; ^3^Department of Biotechnology, Isfahan Pharmaceutical Sciences Research Center, Faculty of Pharmacy, Isfahan University of Medical Sciences, Isfahan 81746-73461, Iran

## Abstract

Targeted drug delivery using folate receptors is one of the most interesting chemotherapeutic research areas over the past few years. A novel folate targeted copolymer was synthesized using dextran stearate coupled to folic acid. FT-IR and NMR spectroscopy were used to confirm successful conjugation. Micelles prepared using this copolymer were characterized for their particle size, zeta potential, critical micelle concentration (CMC), drug loading capacity, and release efficiency. Cytotoxicity and cellular uptake of the micelles were estimated using CT-26 colorectal carcinoma cell line. FT-IR and NMR spectroscopy confirmed production of folate grafted dextran stearate copolymer. Low CMC value indicates that the copolymers are suitable for preparation of stable micelles useful in parenteral dosage forms. Particle size and zeta potential of the targeted nanoparticles were 105.5 ± 2.0 nm and −21.2 mV, respectively. IC_50_ of etoposide loaded in folate grafted dextran stearate enhanced about 20-fold compared to the pure drug (0.49 ± 0.11 **μ**g/mL versus 9.41 ± 0.52 **μ**g/mL). It seems that etoposide loaded in micelles of folate grafted dextran stearate copolymer is promising in reducing drug resistance of colorectal cancer by boosting etoposide cellular uptake.

## 1. Introduction

Colorectal cancer (CRC) contributes to over one million new cases annually and accounts for over one thousand deaths. CRC is the second most common cause of cancer mortality. Approximately CRC comprises 9% of the global cancer burden [1].

There are limited drug therapy choices for management of CRC. Many of these intervention therapies include exclusively 5-FU or 5-FU along with irinotecan, oxaliplatin, panitumumab, cetuximab, or bevacizumab. Despite using these combinations many CRC cases are drug resistant and the lack of novel remedies is sensible [2, 3].

Recently, nanocarriers have been studied to increase cellular uptake of anticancer drugs like etoposide [4]. In fact, such carriers act as drug reservoirs and are capable of providing considerably higher concentration of the drug outside and inside the malignant cells especially after being uptaken, since higher concentration correlates with earlier and more rapid apoptosis induction [4, 5].

Reddy et al. [6] studied etoposide-containing tripalmitin nanoparticles and suggested that the prepared positively charged solid lipid nanoparticles (SLNs) had prolonged residence time in blood circulation. They used these SLNs to treat Dalton's lymphoma tumor bearing mice and found their meritorious effectiveness in tumor uptake [7].

Although most cases of CRC are etoposide resistant, polymeric micelles of etoposide have shown promising results in CT-26 colorectal adenocarcinoma cell line and tumor bearing Balb/c mice [8].

Another reason for the successful approach of using micelles in etoposide delivery especially in animal studies may be due to the enhanced permeation and retention effect (EPR) in transmission of these particles from extraordinarily porous tumor capillaries. Further method for the targeting of nanoparticles to tumor cells is the attachment of homing devices on the surface of nanoparticles that cause their active transport to cells by receptor mediated transcytosis [9]. The most frequently used targeting ligand in cancer therapy is folic acid which targets folate receptors. These receptors include 3 subtypes (*α*, *β*, and *γ*). Observations on CRC patients affirmed overexpression of *α*-folate receptors on cancerous cells [10, 11].

Polymeric micelles consisting of amphiphilic diblock copolymers have been the subject of many studies for the past two decades. Main interest in the field of drug delivery has been specifically focused on the application of micelles in drug solubilization, controlled drug release, and drug targeting [12].

Dextran is a natural branched hydrophilic polymer that is composed of glucose monomers [13]. Stearate grafted dextran has been synthesized by Du et al. [14] via an esterification reaction and is used to carry doxorubicin to nude mice bearing A549 human lung adenocarcinoma. The effect of molecular weight and molar ratio of dextran on self-assembly of dextran stearate polymeric micelles was further studied by Varshosaz et al. [15]. *In vitro* cytotoxicity tests showed good toxic effect against drug-sensitive tumor cells while the polymer itself was nontoxic [15]. Moreover, these micelles presented reversal activity against doxorubicin-resistant cells.

In the present work, we aimed to prepare a novel folic acid targeted dextran stearate polymeric micelle for delivery of etoposide and the evaluation of cytotoxicity has on CT-26 cell line of colorectal cancer. This cell line was chosen as it overexpresses the folate receptors and has been used by Benns et al. [16] for targeted gene delivery using folate-PEG-folate-graft-polyethylenimine copolymer.

## 2. Materials and Methods

### 2.1. Materials

Dextran Mw of 6000 and 10000, stearoyl chloride, pyrene, and dialysis tube with cutoff 2 kDa were purchased from Sigma (USA); folic acid, dicyclohexylcarbodiimide (DCC), dimethyl sulfoxide (DMSO), dimethylformamide (DMF), dimethyl amino pyridine (DMAP), and 3-4,5-dimethylthiazol-2-yl-2,5-diphenyl tetrazolium bromide (MTT) was from Merck Chemical Company (Germany). Etoposide was a gift from Nippon Kayaku Co., Ltd. (Tokyo, Japan). RPMI1640, folate-free RPMI1640 medium, and FBS were supplied from Gibco Laboratories (USA).

### 2.2. Synthesis of Dextran Stearate (DS)

The method reported in our previous work was used for this synthesis [15]. Briefly, 1 g of dextran molecular weight (Mw) 6000 (0.166 mmol) was added to 20 mL DMF and stirred at 120°C for 2 h under nitrogen atmosphere. After cooling the slurry to 80°C, 2 g of LiCl was added and stirred at room temperature for 10 minutes to dissolve completely. Then 180 mg (1.25 mmol) DMAP was added to activate the hydroxyl groups of dextran for further nucleophilic attack to carbonyl group of stearoyl chloride. Subsequently, a solution of stearoyl chloride 420 *μ*L (1.25 mmol) in 20 mL of DMF was added dropwise in 1 h at 80°C and the mixture was stirred for 24 hours. Ethanol (200 mL) was added portionwise to the reaction mixture to precipitate the final compound as pale yellow paste which was collected by filtration and washed with ethanol prior to the dryness in vacuum oven.

### 2.3. Synthesis of Folate Grafted Dextran Stearate (FDS)

Briefly, 67 mg (0.15 mmol) of folic acid and 400 mg of dextran stearate were dissolved in 20 mL DMSO. When a clear solution was obtained 22 mg (0.15 mmol) of DMPA and 31 mg (0.15 mmol) of DCC were added and allowed to react for 72 hours in room temperature. Dicyclohexylurea was removed by filtration. The product was dialyzed using membrane tubing (cutoff 2 kDa) against deionized water for 48 h. Water was exchanged every 12 hours with fresh distilled water and the product was lyophilized.

### 2.4. Determination of Critical Micellar Concentration (CMC)

Fluorescence spectroscopy using pyrene as a hydrophobic fluorescence probe was used to determine CMC of the dextran stearate and folate grafted dextran stearate copolymers. Serial dilution of copolymers between 100 *μ*g/mL and 0.2 *μ*g/mL was done. Then 1 mL of acetone solution of pyrene (6 × 10^−6 ^mol/L) was added to all copolymeric solutions so that the final concentration of pyrene was 6 × 10^−7 ^mol/L. Fluorescence intensity in *λ*_em_ = 390 nm at *λ*_ex_ = 335 and 338 nm was determined using a spectrofluorometer (LS-3, Perkin Elmer, USA).

### 2.5. Analytical Method of Etoposide Detection

Etoposide concentration in micelles was measured using an isocratic HPLC method developed and validated in our laboratory according to a previously published method with a minor modification [17]. The method was validated for linearity, accuracy, and precision. A C18-NUCLEODUR column (MACHEREY-NAGEL, Germany) (particle size 5 *μ*m, 4.6 mm × 250 mm) was used. The mobile phase consisted of acetonitrile/disodium hydrogen phosphate (0.1 M) (35 : 65) with final pH adjusted to 5.2 ± 0.1 with orthophosphoric acid. The mobile phase was eluted at the flow rate of 1 mL/min, and the effluent was monitored at 210 nm using a UV detector. Column temperature was kept at 40°C, and 20 *μ*L of the sample was injected into the HPLC column. Data analysis and processing were performed by millennium software.

### 2.6. Preparation of Etoposide Loaded Copolymeric Micelles and Drug Loading Determination

Twenty mg of etoposide and 40 mg of DS or FDS copolymer were dissolved in 5 mL of DMSO. The DMSO was removed by dialysis in 1 L of acetate buffer (pH 5.5) for 2 hours, followed by dialysis in 1 L of distilled water up to 12 hours. The resulting dispersion of micelles was freeze-dried. Blank micelles were prepared by a similar process but without etoposide. For estimation of entrapment efficiency, 5 mg of drug loaded micelles was dissolved in 10 mL of DMSO in the volumetric flask and 1 mL of methanol was added to 100 *μ*L of this solution. The resulting suspension was centrifuged. The supernatant was separated and dried under nitrogen. Then the residue was reconstituted with 50 mL of mobile phase and measured by the HPLC method described above.

The loading efficiency and loading percentage were calculated using (1) and (2), respectively:

(1) Loading  efficiency (%)=(Analyzed  weight  of  drug  in  micellesTheoretical  weight  of  drug  loaded  in  system)×100,

(2) Loading % =(Analyzed  weight  of  drug  in  micellesTheoretical  weight  of  micelles)×100.

### 2.7. Determination of Size and Zeta Potential of Micelles

Size and zeta potential of micelles were evaluated by dynamic laser light scattering (DLS) method using a Zetasizer (ZEN3600, Malvern Instruments Ltd., UK). Five mg of freeze-dried micelles was redispersed in 5 mL of deionized water and analyzed at a temperature of 25°C and a scattering angle of 90°.

### 2.8. Morphology of Micelles

Transmission electron microscopy (TEM) (LEO 906 E, Germany) was used to study the morphology of the micelles. A drop of drug loaded micelle dispersion of folate grafted dextran stearate copolymer containing 0.01 wt% phosphotungstic acid was applied on carbon coated cupper grid and dried at room temperature for 30 min. After the drop was dried, the shape and size of the micelles were observed with a TEM.

### 2.9. *In Vitro* Drug Release

The dispersion of 5 mg etoposide loaded in DS or FDS polymeric micelles was prepared in 1 mL of phosphate buffered saline (PBS) (0.1 M, pH 7.4). The dispersions were added to the dialysis tubes (cutoff 2 kDa); the tubes were placed in 100 mL of PBS and incubated at 37°C separately. Samples were collected at predetermined time intervals up to 48 h and analyzed with a UV spectrophotometer (UV-mini 1240, Shimadzu, Kyoto, Japan) at *λ*_max⁡_ = 284 nm.

### 2.10. Cellular Uptake Studies

Fluorescein loaded micelles were prepared by solubilizing 40 mg of sodium fluorescein and 40 mg of DS or FDS copolymer in 5 mL of DMSO then dialyzing the solution against 1 L of acetate buffer (pH 5.5) for 12 hours in dialysis tubing with cutoff 2 kDa. The buffer was then exchanged with distilled water and dialysis was continued for 24 h. The resulting dispersion was freeze-dried.

A sterile glass cover was dropped into each well of a 6-well culture plate. 1 × 10^5^ cells of CT-26 cell line in folate-free RPMI1640 were seeded into each well and incubated for 24 h at 37°C. 100 *μ*g of sodium fluorescein or 200 *μ*g of fluorescein loaded DS or FDS micelles was added to each well. After 2-hour incubation at 37°C the cells were washed three times with PBS. A fluorescent microscope (CETI, Belgium) was used for cell imaging.

For quantitative study of cellular uptake flow cytometry method was used. 1 × 10^6^ CT-26 cells in folate-free RPMI1640 were seeded into a 6-well cell culture plate and allowed to grow overnight. 100 *μ*g of sodium fluorescein or 200 *μ*g of fluorescein loaded DS or FDS micelles or 200 *μ*g of blank DS or FDS modified polymer was added to the wells. After 2 h, cells were harvested and washed with PBS and then were studied with a flow cytometer (BD FACSCalibur, USA). The cell uptake ratio was calculated from the dot plot graph of forward scattering versus fluorescence intensity.

### 2.11. *In Vitro* Cytotoxicity Test of Etoposide Loaded Micelles

Dimethylthiazoldiphenyltetrazolium (MTT) assay was performed to estimate *in vitro* cytotoxicity and the IC_50_ of each formulation. CT-26 colorectal cancer cells were seeded in 96-well plates a day prior to the treatment with the density of 2000 cells/well. After 72-hour incubation with different concentrations of blank or etoposide loaded micelles in a humidified 37°C and 5% CO_2_ incubator, 20 *μ*L of 5% aqueous solution of MTT was added to wells and the plates were incubated for 3 hours. Formazan crystals were separated and solubilized in 150 *μ*L of DMSO. The absorbance of each sample was measured at 570 nm using an automated microplate reader (Awareness, USA).

## 3. Results and Discussion

### 3.1. Synthesis of Folate Grafted Dextran Stearate

**Figure 1:**
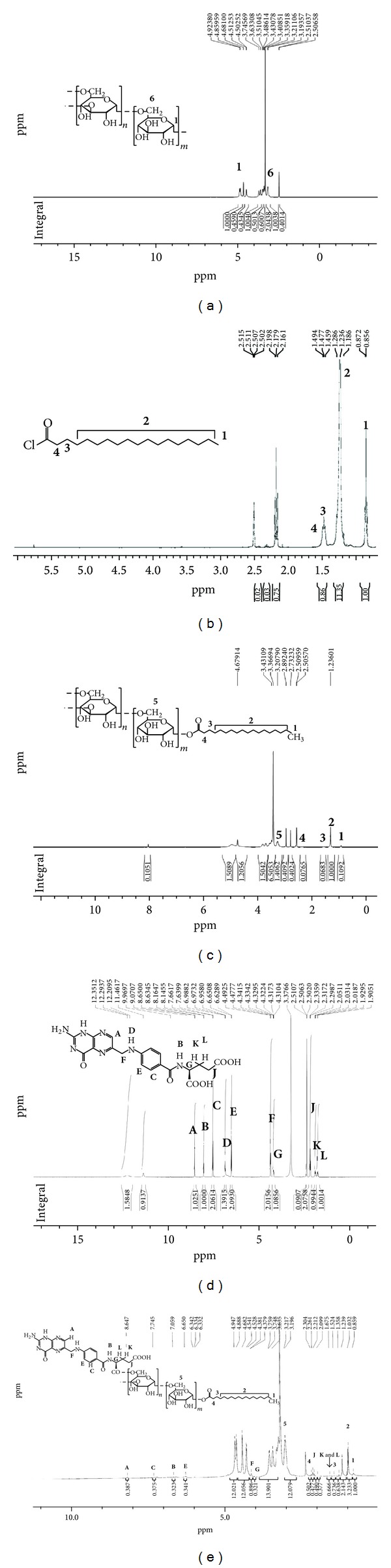
The ^1^H NMR spectra of (a) dextran 6000, (b) stearoyl chloride, (c) dextran-stearate copolymer, (d) folic acid, and (e) folate-dextran stearate copolymer.

**Figure 2:**
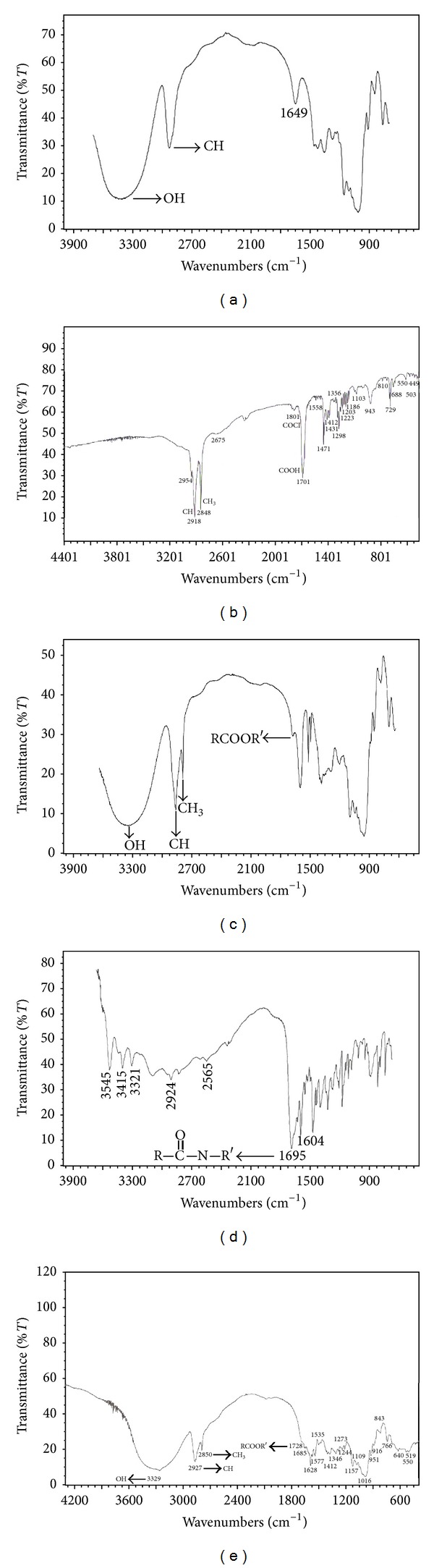
The FTIR spectra of (a) dextran 6000, (b) stearoyl chloride, (c) dextran stearate copolymer, (d) folic acid, and (e) folate-dextran stearate copolymer.

Dextran stearate was synthesized by reaction of acyl chloride. Stearoyl chloride is an unstable reagent and rapidly reacts with moisture of the air and solvents used for product purification and consequently it is converted to stearic acid. Therefore, there are some limitations to take FTIR spectrum of stearoyl chloride as it is rapidly converted to stearic acid. However, the FTIR and NMR spectra of this chemical are shown.  Figure 1 shows the ^1^H NMR spectra of dextran 6000 (Figure 1(a)), stearoyl chloride (Figure 1(b)), dextran stearate copolymer (Figure 1(c)), folic acid (Figure 1(d)), and folate-dextran stearate copolymer (Figure 1(e)). In ^1^H NMR spectrum of stearoyl chloride, the nearest protons to carbonyl group signals are observed at 1.46 and 2.161 ppm (Figure 1(b)). In dextran stearate ^1^H NMR spectrum (Figure 1(c)), these signals are shifted slightly downfield as is expected and is observed at 1.50 and 2.31 ppm, respectively. These signals were shifted to higher domains (*δ* ppm) due to the deshielding effect of esteric bond formation on adjuvant methylene groups in the product (Figure 1(e)). Moreover, FTIR results seen in Figure 2 show that the signal related to carbonyl group of stearoyl chloride (Figure 2(b)) seen at 1801 and 1701 cm^−1^ (due to rapid change of stearoyl chloride to stearic acid in the atmospheric conditions) is shifted to 1747 cm^−1^ in the final product (Figure 2(e)) and confirms the formation of esteric bond.

In ^1^H NMR spectrum of folic acid, protons between two carbonyl group signals are observed aside in 1.905 and 2.031 ppms (Figure 1(d)). ^1^H NMR spectrum of final compound (folate grafted dextran stearate) (Figure 1(e)) includes a peak in 1.671 ppm that refers to the two nearest hydrogen atoms to carbonyl group. Also, appearance of weak signals at 6.7–8.7 ppm, which corresponded to the aromatic protons of folic acid, confirms the presence of folic acid in the final product. The terminal CH_2_ peaks of folic acid appeared at 2.304 ppm. Another reason for this conjugation is interpreted from the FTIR results. So in the FTIR spectrum of the product (Figure 2(e)) a new signal related to the stretching vibration of the esteric carbonyl group of folic acid (Figure 2(d)) was added to 1697 cm^−1^ which is seen in 1728 cm^−1^.

The degree of substitution of stearic acid per each glucose unit of dextran was calculated from ^1^H NMR spectrum of the final product (Figure 1(e)). The signals of anomeric proton of dextran are observed in *δ* = 4.849 ppm and are clearly distant from other signals. CH_3_ of stearate branches is observed in upper field of NMR spectrum (*δ* = 0.859 ppm) (see (3)). The areas under the curve of these two peaks were used to estimate the degree of substitution. Based on this definition the degree of substitution of stearate branches per each glucose unit of dextran was calculated and the results are represented in Table 1:

(3) Degree  of  substitution  of  stearic  acid  =Area  under  peak  δ0.8593×Area  under  peak  δ4.849×100,

(4) Degree  of  substitution  of  folic  acid  =Area  under  peak  δ8.647Area  under  peak  δ4.849×100.

The degree of substitution of folic acid on folate-dextran stearate copolymer (Figure 1(e)) was calculated as ratio of the last aromatic peak of folic acid (*δ* = 8.647) and the anomeric proton of dextran observed in *δ* = 4.84976 ppm (see (4)). Based on these calculations, the degree of substitution of folic acid per each dextran's glucose unit is represented in Table 1.

### 3.2. Critical Micellar Concentration (CMC)

There are different methods for estimation of CMC by pyrene. Below the CMC, pyrene is solubilized in water, which is a highly polar medium. When micelles are formed, pyrene partitions preferentially toward the hydrophobic domain afforded by the core of micelle. Thus, a change in the vibrational fine structure of the emission spectra and a red shift of the (0,0) band in the excitation spectra are observed. The apparent CMC can be obtained from the plot of the fluorescence of pyrene, the *I*_1_/*I*_3_ ratio from emission spectra, or the *I*_335_/*I*_338_ ratio from excitation spectra, against concentration; a major change in the slope indicates the onset of micellization [18]. In recent literatures, more accurate results have been achieved by monitoring changes in ratio of the pyrene excitation intensity spectra. In this research, we also used a fixed emission wavelength of *λ*_em_ = 390 nm and the excitation intensities of third energy band (*I*_3_) were obtained for pyrene [19].

**Figure 3:**
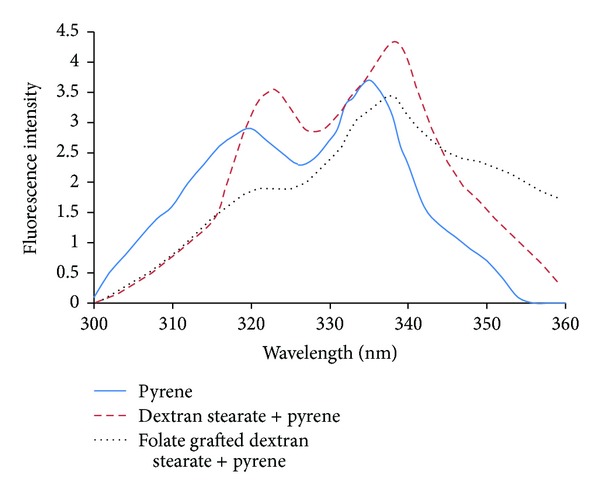
Excitation spectra of pure pyrene or pyrene in the presence of modified copolymers.

Based on the above spectra, maximum fluorescence intensities' third energy band (*I*_3_) (see Figure 3) for pyrene in a hydrophobic medium (modified copolymers) was *λ*_ex_ = 338 nm while for pure pyrene in water it was 335 nm.

**Figure 4:**
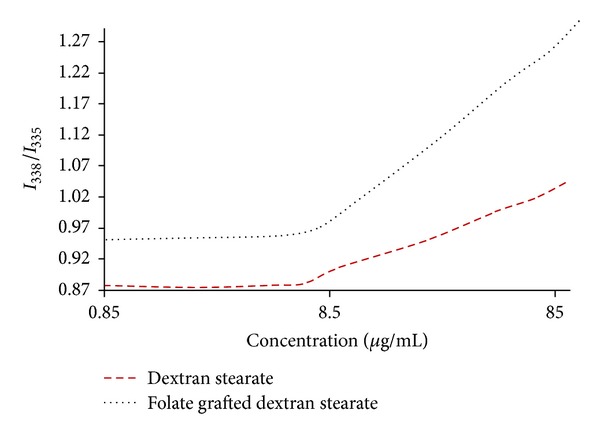
Changes of fluorescence intensity ratio (*I*_338_/*I*_335_) versus log of copolymers concentration.

According to Figure 2 the CMC can be estimated from the intersection of the horizontal line with an almost constant value of the ratio of *I*_338_/*I*_335_ (4 beginning points on both curves in Figure 4) and the vertical line with a steady increase of this ratio (5 terminal points on both curves in Figure 4). The CMC value for each modified dextran is represented in Table 1.

In the aqueous solvents, the hydrophobic stearic acid moieties of the DS or FDS were assembled in hydrophobic cores, while these cores were surrounded by hydrophilic dextran backbones, to achieve the lowest Gibbs free energy level. The spontaneous self-aggregation of the DS and FDS in the aqueous medium was determined by the dye solubilization method using pyrene as the probe molecule. The emission spectra of pyrene depend strongly on the polarity of the microenvironment; in polar solvents, the intensity of the first energy band (338 nm, *I*_1_) of the pyrene emission spectrum is higher than that of the third (338 nm, *I*_3_) whereas in a hydrophobic environment, *I*_3_ is higher than *I*_1_. Therefore, when micelles are formed in an aqueous medium, the pyrene tends to locate itself inside the hydrophobic core, increasing *I*_3_ intensity. As a result, the ratio of *I*_1_ to *I*_3_ can be used to determine the CMC.

Basic practical application of CMC in polymeric micelles is estimation stability of micelles during storage or even after i.v. administration and dilation in blood; as a consequence, they are expected to be disassociated. Thus, the lower the CMC value, the more stable the micelles in the body fluids [20]. At concentrations lower than the CMC, the ratios were approximately the same, whereas in concentrations higher than the CMC, a linear increase in the ratios was observed with increasing DS and FDS micelles concentration. The low CMC values indicate their ability to form stable micelles and keep their intact structure upon dilution with body fluids. Nayebsadrian et al. [21] reported the CMC value of 15 *μ*g/mL for retinoic acid grafted dextran micelles, while the CMC reported for retinoic acid grafted chitosan micelles was 13.5 *μ*g/mL [22], around 155 and 170 times respectively, lower than the concentrations needed to form micelles with low-molecular-weight surfactants, for example, sodium dodecyl sulphate (SDS), which has a CMC of 2.3 mg/mL. These results clearly indicate that the DS and FDS copolymers can form micelles even in highly diluted solutions and generally the copolymeric micelles show much higher capacity for the formation of stable micelles than the low-molecular-weight surfactants.

### 3.3. Physical Characteristics of Micelles

Physical properties of etoposide loaded micelles including particle size, zeta potential, and drug loading efficacy are presented in Table 2.

Dialysis method was used to load the drug in the copolymeric micelles. Usually this method is used for copolymers which cannot be solved in volatile organic solvents. The other advantage of this method is the uniformity of micelles in size and distribution. As Table 2 shows both untargeted (DS) and targeted micelles (FDS) had acceptable particle size of 169 and 105 nm, respectively, and low polydispersity index (PdI) of around 0.3 which indicates low diversity and particle size distribution of the micelles. Considering that the PdI is calculated from the square of the standard deviation/mean diameter, less value of PdI indicates enhanced homogeneity of the nanoparticles [23]. The suitable PdI of 0.2 is also reported in many works on nanoparticulate drug delivery systems like in PLGA nanoparticles of paclitaxel [23] and chitosan nanoparticles cross-linked with TPP [24].

Zeta potential is a function of surface charge of particles. Therefore, if the absolute value of zeta potential is greater, micelles stability toward aggregation will be superior. The results of Table 2 indicate higher negative values for zeta potential in FDS than in DS. The possible reason may be due to the carboxylate groups of folate and stearic acid conjugated groups in folate targeted micelles and hence higher stability for these micelles is expected. Müller et al. [25] have reported that potentials between −5 and −15 mV are in the region of limited flocculation and between −5 and −3 mV of maximum flocculation in nanoparticles [25, 26]. Moreover, another reason for higher zeta potential of FDS micelles compared to DS is due to the larger particle size of DS micelles (Table 2) which can result in the reduction of charge density and decrease the absolute zeta potential of these micelles.

It is concluded from Table 2 that by adding targeting ligand to particles both drug loading percent and efficacy have been decreased significantly (*P* < 0.05). This decrease in drug loading in targeted micelles may be due to repulsion of the more negative micelles of FDS with the drug molecules which have a negative charge and decrease in the loading efficiency.

**Figure 5:**
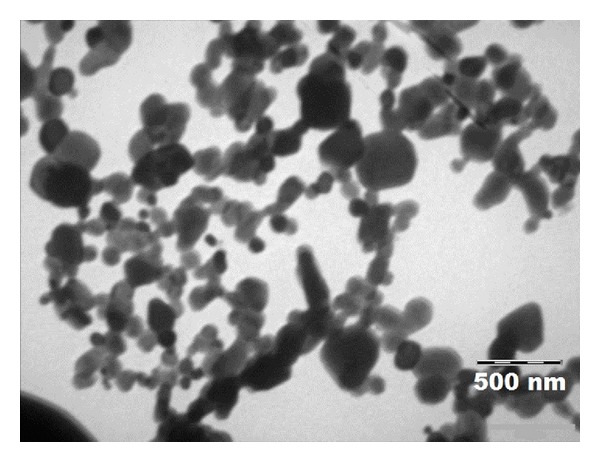
TEM photographs of etoposide loaded folate targeted dextran stearate polymeric micelles.

The morphology of etoposide loaded folate targeted polymeric micelles was studied by transmission electron microscopy (TEM). As shown in Figure 5, the FDS copolymer had spherical shape particles in the aqueous solution. Although some aggregates of the micelles were obvious, many discrete spherical particles were present. The scale bar of the graphs confirmed the particle size of the micelles obtained by DLS method (Table 2).

### 3.4. *In Vitro* Drug Release

**Figure 6:**
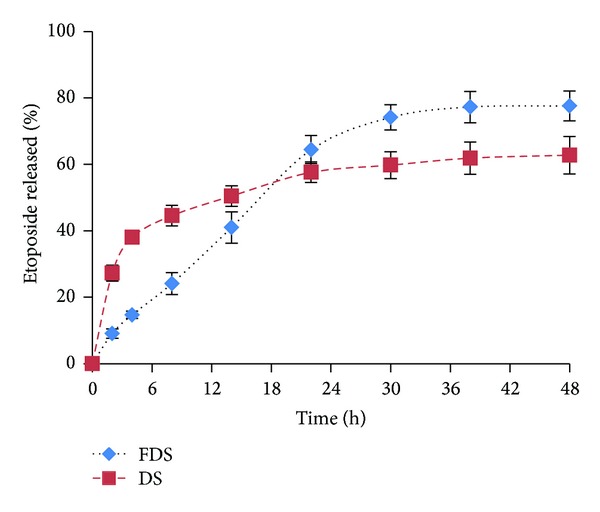
Etoposide release profiles from dextran stearate (DS) and folate targeted dextran stearate (FDS) polymeric micelles in phosphate buffered saline (pH 7.4) (*n* = 3).

Figure 6 shows etoposide release profiles from drug loaded FDS and DS polymeric micelles in phosphate buffered saline (PBS; 0.1 M, pH 7.4). Etoposide release from FDS polymer showed a near zero order kinetic till 24 h of release test and had lower initial burst release than DS polymeric micelles. Burst release of etoposide from DS micelles is seen in Figure 6 (about 40% in 4 hours) and saturation in drug release happened beyond 20 hours while FDS micelles had low initial release and saturation in drug release occurred only beyond 36 hours. This could be due to the greater amount of drug loaded in DS micelles (Table 2) and consequently the higher drug concentration gradient in these micelles. Moreover, the superficial drug loading between the interface of the core and corona of the DS micelles may be the reason of the burst release of the drug; while in the FDS micelles possibly the drug was more loaded in the hydrophobic core of the micelles due to the presence of folic acid which caused the steric hindrance for approaching the drug to the corona and better accommodation of the drug in the core of the micelles which delayed the drug release and lack of the burst effect.

Table 2 also shows the release efficiency (RE_48_%) values. As can be seen in Figure 6 and Table 2, the percentage of drug released was enhanced in the case of folate coupled micelles. For example, in the case of DS micelles drug release was found to be 62.7 ± 5.6% at the end of 48 h and the calculated RE_48_% value was 51.15 ± 1.32%, whereas in the case of FDS drug release percent was found to be 77.6 ± 4.4% and its RE_48_% was 57.08 ± 2.37%. Thus, drug release from the micelles is being affected by folate conjugation. This is contrary to the obtained results for transferrin (Tf) coupled liposomes; as Soni et al. [27] reported, Tf coupling provides a structural integrity, which terminates in a double barrier effect for drug diffusion.

Differences between the particle sizes of the nanoparticles (Table 2) can be considered as the possible reason for the variation of release profiles which can explain the observed differences between FDS and DS micelles. Since smaller particle sizes have shorter drug diffusion distance and larger surface area, they show faster drug release [28].

Hossann and coworkers [29] have recently investigated the influence of the size of thermosensitive liposomes on their content release in broad terms and revealed that even small changes in the particle size impact the release properties of the particles.

Albeit the differences of the particle sizes can fully explain the different release diagrams, the interactions between the drug and polymer molecules must also be mentioned as the second explanation since the physicochemical properties of the drug have a considerable effect on the rate and the control of drug release [30]. In brief, the lower tendency of etoposide molecules to negatively charged micelles of FDS is responsible for the higher release rate and amount of drug released. By contrast, the electrostatic repulsion of etoposide to FDS micelles results in the easier and consequently faster drug diffusion from the targeted micelles.

Drug release from DS in Figure 6 shows a two-phase release pattern with an initial burst release and a subsequent more sustained release of the entrapped drug. Amiodarone loaded lipid nanocapsules were shown to have similar release profiles due to the accumulation of the drug near the nanoparticle surface [31]. However, drug release from FDS was more gradual in the first 24 h and nearly a zero order release kinetic was observed so that it took 2 days for the release of 77% of the drug (Figure 6). This slow and prolonged release is attributable to the hydrophobic nature of stearate and perhaps as well to the crystallization of drug inside the micelle's core [32, 33]. Considering that the micelles retained the drug inside the core due to the slow drug leakage, it can be concluded that the drug is released only when the micelles are taken up and the dextran molecules are enzymatically degraded within the tumoral cells.

### 3.5. Cellular Uptake Studies

Quantitative uptake of particles in CT-26 cell line was determined by flow cytometry method, and fluorescent microscopy was used for qualitative observation of cellular uptake of micelles.

Sodium fluorescein was applied as a fluorescent label in these studies. Previous studies showed that this soluble salt of fluorescein cannot be uptaken by cells until metabolism of cells makes the culture media acidic [34].

**Figure 7:**
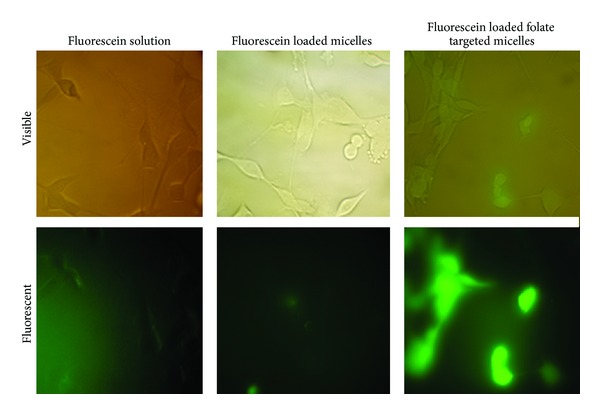
Images taken by fluorescent and visible light microscopes of CT-26 cells after incubation with fluorescein loaded micelles or fluorescein solution for 2 h.

It is concluded from Figure 7 that the majority of cells incubated with sodium fluorescein loaded in FDS micelles had green fluorescence, while only some of the cells incubated with labeled DS micelles had weak green fluorescence. The background of cells treated with sodium fluorescein solution was green in fluorescent image, and the dark shadows of cells in this part of Figure 7 are obvious. Loading efficiency of sodium fluorescein in FDS and DS was measured by UV-spectrophotometric method in 490 nm and was 51.11 ± 1.23% and 48.65 ± 0.78% for FDS and DS micelles, respectively. It is well established that polymeric micelles can be loaded by hydrophobic, hydrophilic, and amphiphilic drugs. After 2 hours the total amount of fluorescein released was measured to be about 10% for both types of the micelles. In other words, it seems that most of the fluorescent probe is still trapped in the micelles during the cellular uptake test.

**Figure 8:**
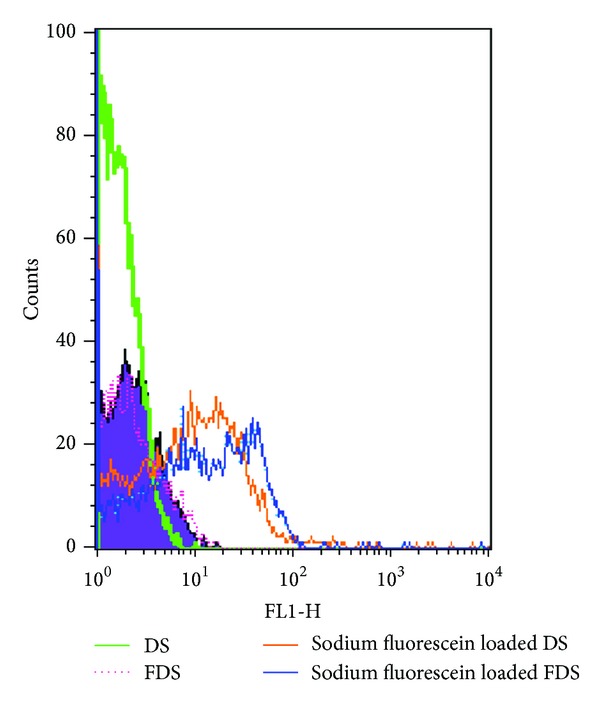
Flow cytometry histogram profiles of CT-26 cells incubated with free sodium fluorescein, sodium fluorescein loaded FDS, sodium fluorescein loaded DS micelles, free FDS, and free DS for 120 min.

For quantitative uptake comparison flow cytometry test was performed on sodium fluorescein, sodium fluorescein loaded DS micelles, fluorescein loaded FDS micelles, blank DS, and blank FDS. As it is obvious from Figure 8, sodium fluorescein loaded FDS had maximum uptake. Nearly all of the cells incubated with blank DS, blank FDS, and free sodium fluorescein had fluorescence intensity less than 10. By gating this part of cells, the percentage of cells that had fluorescence intensity more than 10 was obtained. More than 66% of counted cells which were incubated with FDS loaded micelles had green fluorescence, while in the cells incubated with DS micelles containing sodium fluorescein, this value was 50.27% which indicates higher cellular uptake of folate targeted micelles into the cells.

### 3.6. Cytotoxicity Assays

**Figure 9:**
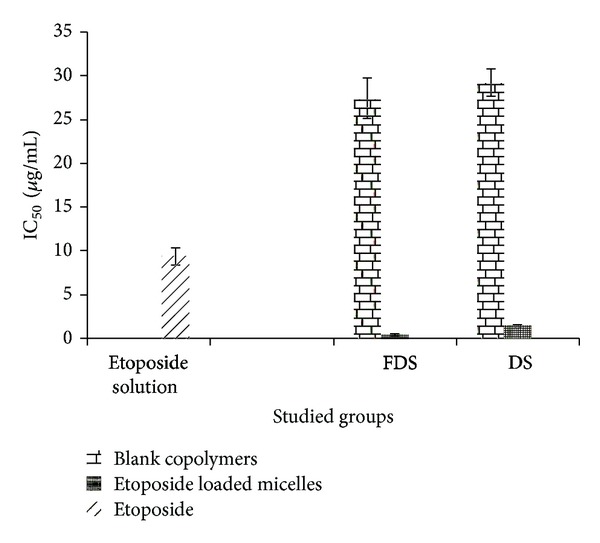
IC_50_ of free etoposide, DS blank copolymer, FDS blank copolymer, etoposide loaded DS micelles, and etoposide loaded FDS micelles.

In the MTT assay, a mitochondrial dehydrogenation enzyme in viable cells cleaves the tetrazolium rings of the pale yellow MTT and forms formazan crystals with a purple color [35].  Figure 9 shows the IC_50_ of etoposide, blank copolymers, and etoposide loaded micelles. As shown in this figure, the IC_50_ of blank copolymers is significantly higher than free etoposide and lower than etoposide loaded copolymeric micelles (*P* < 0.05). There is no significant difference between cytotoxicity of two blank copolymers of DS or FDS. IC_50_ of etoposide loaded FDS micelles is significantly lower than free etoposide and etoposide loaded DS micelles (*P* < 0.05). These observations implied that etoposide loaded FDS micelles were more cytotoxic than etoposide loaded DS micelles. Previous studies [4] showed that etoposide lipid nanocapsules showed a generally higher efficiency than the drug solution in cytotoxicity for glioma cell lines. The mechanism of action of etoposide nanocapsules was proposed to be a cell uptake followed by a sustained drug release from the nanocapsules in combination with an intracellular P-gp inhibition ensuring a higher anticancer drug concentration inside the cancer cells.

**Figure 10:**
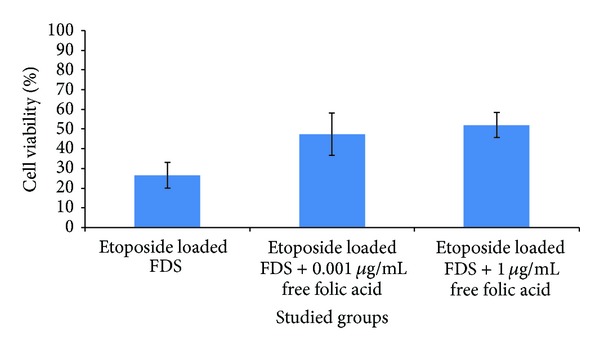
Competitive binding effect of different concentrations of free folic acid on the viability of CT-26 cells incubated with etoposide loaded FDS in folate-free medium (etoposide concentration = 10 *μ*g/mL). Error bars denote standard deviation.

Competitive binding experiments were performed to determine whether folate ligands specifically mediated the cellular uptake of FDS micelles. For this purpose, free folic acid was added to the folate-free culture medium of CT-26 cells in 0.001 and 1 *μ*g/mL concentration and the MTT assay was repeated with the FDS etoposide loaded micelles and free etoposide. Addition of free folic acid to the culture medium caused 26.5 ± 6.5% cell viability in FDS drug loaded micelles compared to 51.98 ± 6.3% in FDS loaded drug micelles in the presence of 1 *μ*g/mL of free folic acid (Figure 10). This enhancement in cell viability to about 2-fold reinforces the conclusion that greater cellular uptake and cytotoxicity of etoposide loaded FDS in comparison to etoposide loaded DS are most likely due to the folate receptor mediated endocytosis of FDS copolymers.

In the study of Saul et al. [36], KB cells also showed a 78% reduction in the amount of cell-associated folate targeted liposomal doxorubicin when incubated with 4 *μ*g/mL (1 mM) free folic acid. The high concentration of free folic acid necessary to competitively inhibit folate receptor-dependent binding and uptake has previously been postulated to be due to the multiple interactions between folate receptors and folate targeting ligands on the liposome, which leads to an exponential effect on the binding affinity between the ligand and receptor.

## 4. Conclusion

Folate grafted dextran stearate was synthesized successfully. This copolymer could produce stable nanosized micelles, which encapsulated etoposide inside their hydrophobic core and controlled the drug release for more than 2 days. Micellization of etoposide in FDS increased cellular uptake of the drug. This study also indicates that cells bearing folate receptors show saturable effect in etoposide uptake as a function of folate targeting ligands. Enhanced cellular uptake of this drug via folate receptors endocytosis and the reduction in the IC_50_ of etoposide loaded in the FDS micelles is hoped to consequently slake multidrug resistance significantly. Most likely, mechanism of this resistance attenuation is augmentation of cellular uptake by folate receptor mediated endocytosis.

## Figures and Tables

**Table 1:** Properties of different synthesized copolymers.

Copolymer type	Degree of substitution of stearic acid	Degree of substitution of folic acid	CMC *μ*g/mL
Dextran stearate (DS)	2.4%	—	7.44 ± 0.25
Folate-dextran stearate (FDS)	2.77%	3.21%	6.67 ± 0.36

**Table 2:** Physical characteristics of etoposide loaded in DS and FDS copolymeric micelles.

Copolymer	Particle size (nm)	Polydisparity index	Zeta potential mV	Drug loading %	Drug loading efficiency %	Release efficiency % until 48 h
Dextran stearate	169.6 ± 14.1	0.363 ± 0.002	−19.2	31.7 ± 0.8	93.3 ± 2.4	51.15 ± 1.32
Folate-dextran stearate	105.5 ± 2.0	0.350 ± 0.015	−21.2	28.4 ± 3.0	82.3 ± 2.5	57.08 ± 2.37
